# An audit of patient radiation doses in interventional radiology at a South African hospital

**DOI:** 10.4102/sajr.v27i1.2559

**Published:** 2023-01-19

**Authors:** Oneile Slave, Nasreen Mahomed

**Affiliations:** 1 Department of Radiology, Faculty of Health Sciences, University of the Witwatersrand, Johannesburg, South Africa

**Keywords:** Interventional radiology, diagnostic reference level, kerma air product, reference point air kerma, fluoroscopy time

## Abstract

**Background:**

Interventional radiology (IR) is becoming more relevant in patient care and is associated with increased patient radiation exposure and radiation-induced adverse effects. Diagnostic reference levels (DRLs) are crucial for radiation control. There is a paucity of published DRLs for IR in South Africa and sub-Saharan Africa.

**Objectives:**

This study aimed to determine local DRLs for fluoroscopically-guided IR procedures and compare the achieved DRLs with published local and international DRLs.

**Method:**

Retrospective, descriptive, single-centre study. Kerma air product (KAP), reference point air kerma (K_a,r_) and fluoroscopy time (FT) were collected for patients (12 years and older) who underwent IR procedures at a university hospital from 01 January 2019 to 31 December 2019. The 75th percentile of the distribution of each dose parameter (KAP, K_a,r_ and FT) per procedure was calculated and taken as the local diagnostic reference levels (LDRL). The established LDRLs were compared to published DRLs.

**Results:**

A total of 564 cases were evaluated. The 13 most frequent procedures (with 15 or more cases) represented 86.1% (487/564). Percutaneous transhepatic biliary drainage was the most common procedure (*n* = 146, 25.9%). Diagnostic cerebral angiogram DRLs exceeded the published DRL data ranges for all parameters (DRL 209.3), and interventional cerebral angiogram exceeded published ranges (DRL 275). Uterine artery embolisation (UAE) exceeded these ranges for KAP and K_a,r_. (KAP-954.9 Gy/cm^2^, K_a,r_-2640.8 mGy).

**Conclusion:**

The LDRLs for diagnostic cerebral angiogram, interventional cerebral angiogram and UAE exceeded published international DRL ranges. These procedures require radiation optimisation as recommended by the International Commission on Radiological Protection (ICRP).

**Contribution:**

In addition to informing radiation protection practices at the level of the institution, the established LDRLs contribute towards Regional and National DRLs.

## Introduction

Use of interventional radiology (IR) in patient care is increasing,^1,2^ and in some countries, it is doubling every 2–4 years.^3^ This calls for effective radiation control. Radiation exposure can lead to deterministic (radiation-induced tissue injuries) or stochastic effects. The linear-no-threshold (LNT) model, derived in part from epidemiological studies,^4^ is used to estimate the risk of stochastic effects. Because there is no threshold, all radiation doses are afforded the same scrutiny. There are measures to minimise patient radiation dose in IR.^5^ Limited knowledge and awareness of patient radiation exposure amongst non-radiology doctors^6,7^ and radiologists^8,9^ contributes to suboptimal patient radiation protection in South Africa. This leads to significant underestimation of dose and the risk of adverse events.

Radiation control is achieved through optimisation, justification and dose limitation without compromising image quality.^10^ For optimisation, the International Commission on Radiological Protection (ICRP) recommends the use of diagnostic reference levels (DRLs). A DRL value is set at the 75th percentile of the distribution of a radiation parameter observed in a facility (typical DRLs), a few facilities (local DRLs) or multiple facilities throughout a country (national DRLs).^10^ The DRLs are not set as radiation limits, nor are they meant for individual patients; instead, the median values (from multiple patients undergoing a procedure) of a DRL parameter are compared with typical, local, national or regional values. If the obtained DRL value exceeds these values, an investigation should be undertaken and corrective measures implemented without undue delay. The ICRP acknowledges that establishing DRLs for IR procedures is more complex because of variation in patients (patient anatomy and clinical factors) and the lesions (pathology) being treated.^10^ To account for this, some studies use a complexity factor to normalise DRL values. This requires substantial clinical data that is not always available. Diagnostic reference levels obtained without factoring complexity are of substantial use.

There is limited published data on DRLs in South Africa and sub-Saharan Africa. This is true for low- and middle-income countries (LMICs) in comparison to high-income countries. In 2015, less than one-quarter of the 135 LMICs had any form of published DRL data.^11^ There has, however, been a trend towards an increasing number of publications: 5 in 1997–2006, 18 in 2007–2011 and 30 in 2012–2015.^11^ Very few articles have been published on South African DRLs for diagnostic radiology and even fewer for IR fluoroscopically guided procedures. A 2021 review of South African DRL data^12^ showed that there were only DRL data from three of the nine provinces (a requirement for national DRLs is that data should be from all provinces) and that there were no DRLs established for mammography and dental procedures (not all the five major imaging modalities).

As far as the authors are aware, there are only two articles establishing DRLs for IR in sub-Saharan Africa. The most recent study is from South Africa by Malan et al.^13^ at Stellenbosch University. It was published in 2020 and sought to determine the local diagnostic reference levels (LDRLs) for common fluoroscopically-guided procedures in the South African context and to compare those to published international data. The other study was from Kenya, conducted in 2013 by Korir et al.^14^ to quantify ionising radiation exposure to patients during interventional procedures and establish national diagnostic reference levels (NDRLs) for clinical radiation exposure management.

This study sought to address the paucity of DRL data for IR procedures in South Africa. The aim was to establish local DRLs for fluoroscopically-guided IR procedures and compare the achieved DRL to published local and international DRLs.

## Research methods and design

The study was designed as a retrospective, descriptive, single-centre study. The study population included consecutive patients (adolescents over the age of 11 years and adults) who underwent fluoroscopically-guided IR procedures (diagnostic or therapeutic) at Chris Hani Baragwanath Academic Hospital (CHBAH) from 01 January 2019 to 31 December 2019. The period was chosen to reflect pre-coronavirus disease 2019 (COVID-19) figures. Chris Hani Baragwanath Academic Hospital is located in Soweto in Gauteng, South Africa. It is a tertiary-level 3400-bed (the third largest in the world) hospital and is the main teaching hospital for the University of the Witwatersrand medical school.

### Data collection

The kerma air product (KAP), reference point air kerma (K_a,r_) and fluoroscopy time (FT) were automatically generated by the fluoroscopy unit at the conclusion of each procedure. Radiographers recorded this data in logbooks, from which the researchers acquired it. The department at the time of the study used two fluoroscopy units, the Philips Allura Xper FD20/20 (biplane) and the Philips Allura Xper FD20 (monoplane), which were both installed in 2010.

### Dosimetry

The dose area product (DAP) – indirect dose parameters – was provided by built-in software for the biplane system. The KAP is the integral of air kerma (the energy extracted from an X-ray beam per unit mass of air in a small irradiated air volume; for diagnostic X-rays, the dose delivered to that volume of air) across the entire X-ray beam emitted from the X-ray tube.^15^ Air kerma at the patient reference point (K_a,r_), also known as cumulative dose or reference dose, is the air kerma accumulated at a specific point in space (the patient entrance reference point) relative to the fluoroscopic gantry.^15^ Fluoroscopy time refers to all the time spent using fluoroscopy. This correlates poorly with other dose indicators.^16^

### Data analysis

The distribution of IR procedures in this sample was tabled using the frequency function on Microsoft Excel. The most frequent procedures were identified (15 or more cases) and included in the analysis. Conversely, procedures with fewer than 15 cases were excluded from the analysis. The KAP, K_a,r_ and FT for the included procedures were captured. The mean, median (50th percentile) and 75th percentile of the distribution of each radiation exposure parameter were determined for each procedure. The 75th percentile of data distribution for the DAP and K_a,r_ of each IR procedure was taken as the LDRL. The LDRLs were compared with published local and international DRLs.

### Ethical considerations

Ethical approval to conduct this study was obtained from the University of the Witwatersrand Human Research Ethics Committee (ref. no. M220320).

## Results

### Frequency of interventional radiology procedures

**FIGURE 1:**
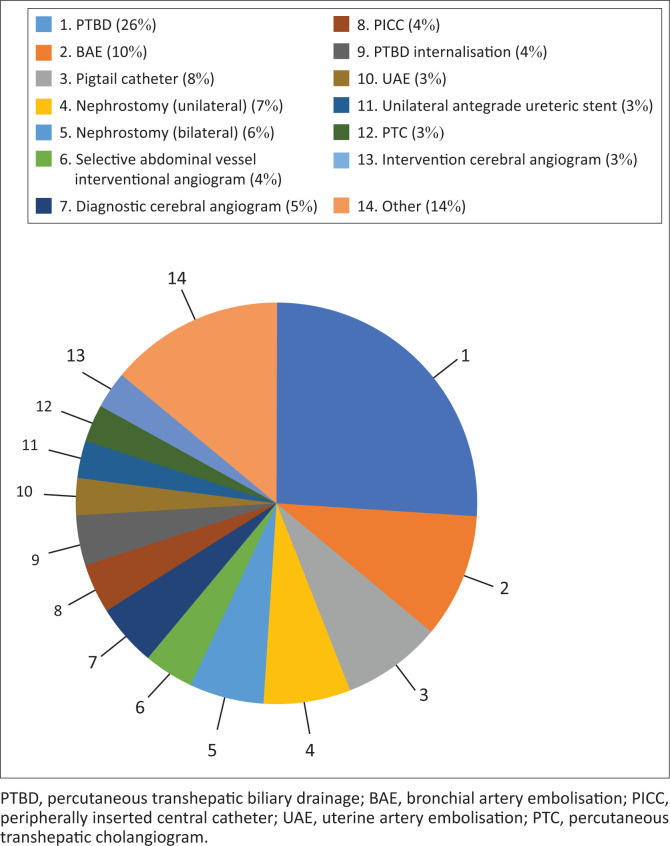
The total number of interventional radiology cases performed during the study period was 564. The 13 most frequent procedures (each with 15 or more cases) representing 86.1 % (*n* = 487) of all cases are reflected.

The total number of IR cases performed during the study period was 564. The 13 most frequent procedures (each with 15 or more cases) represented 86.1% (*n* = 487) of all cases, as reflected in Figure 1. Procedures that had 14 or fewer procedures included transthoracic needle lung biopsy (*n* = 6), transjugular liver biopsy (*n* = 5) and transjugular intrahepatic portosystemic shunt (TIPS) (*n* = 1). The most frequently performed procedure was percutaneous transhepatic biliary drainage (PTBD) (*n* = 146). The biplane angiography unit was nonfunctional for four months (April 2019 – July 2019). During this period, no neuro-interventional cases were conducted, affecting the total number of cases.

### Radiation exposure parameters

Table 1 represents the per procedure median, interquartile range, 75th percentile and 75th:50th ratio for KAP, K_a,r_ and FT. The highest radiation dose was recorded for uterine artery embolisation (UAE) (KAP = 954.9 Gy/cm^2^, K_a,r_ = 2640.8 mGy). The longest FT LDRL was recorded for interventional cerebral angiogram (34.1 min). Peripherally inserted central catheter (PICC) insertion had the lowest LDRL for both KAP (2 Gy/cm^2^) and K_a,r_ (5 mGy). The shortest FT LDRL was for percutaneous transhepatic cholangiogram (PTC) (0.7 min).

**TABLE 1:** Dosimetry data for Chris Hani Baragwanath Academic Hospital.

Procedure	Total cases (n)	Kerma air product (Gy/cm^2^)				Reference point air kerma (mGy)				Fluoroscopy time (min)			
		Median	IQR	75th percentile	75th:50th	Median	IQR	75th percentile	75th:50th	Median	IQR	75th percentile	75th:50th
Percutaneous transhepatic biliary drainage (PTBD)	146	11	19	24	2.2	64	100, 8	131.8	2.1	3.3	4, 5	6.2	1.8
Bronchial artery embolisation (BAE)	57	76	70	131	1.7	215	197	343	1.6	21.5	18, 4	33.5	1.6
Pigtail insertion	44	3.5	6, 5	7.5	2.1	19	31	37	1.9	1.1	1, 8	2.4	2.1
Nephrostomy (unilateral)	42	2	8	10	5	20	28	26	1.8	1.7	2, 6	3.4	2.1
Nephrostomy (bilateral)	37	5.5	7	10	1.8	33	41	62	1.9	3.6	3, 6	6.3	1.8
Selective abdominal vessels-interventional angiogram	26	385	600, 3	776	2	1208	1819, 5	2227.8	1.8	19.7	17, 2	28.3	1.4
Diagnostic cerebral angiogram	26	157.5	117, 3	209.3	1.3	598.5	420, 8	868.5	1.5	18.8	15, 9	28.4	1.5
PICC	25	1	1, 0	2	2	2	4, 0	5	2.5	1.8	3, 2	4	2.2
PTBD internalisation	20	38	32, 3	57	1.5	181.5	166, 8	259	1.4	13.4	9, 25	16.7	1.2
Uterine artery embolisation (UAE)	18	602.5	1071	1463.8	2.4	1339	3070, 8	4019	3	12.3	17, 6	24.8	2
Unilateral antegrade ureteric stent	16	14	17	23	1.6	44	94	118.5	2.7	9.5	11, 5	15.2	1.6
Percutaneous transhepatic cholangiogram (PTC)	15	6	6	9	1.5	21	20, 5	28.5	1.4	0.4	0, 4	0.7	1.8
Interventional cerebral angiogram	15	107	242	275	2.6	1502	987, 5	1744	1.2	26.3	18, 0	34.1	1.3

IQR, interquartile range; PICC, peripherally inserted central catheter.

The ratio of the 75th to 50th centile is used as a measure of the variation with a dose parameter (Table 1). The procedures with the narrowest variation in dose for each parameter were diagnostic cerebral angiogram (1.3) for KAP, interventional cerebral angiogram (1.2) for K_a,r_ and PTBD internalisation (1.2) for FT. Procedures with the widest variation in dose were unilateral nephrostomy (5) for KAP, UAE (3) for K_a,r_ and PICC (2.3) for FT.

### Comparison with published diagnostic reference levels

Tables 2a–c represent CHBAH LDRLs in comparison to published local and international DRLs. There were comprehensive comparable DRLs for 6/13 procedures, namely PTBD, bronchial artery embolisation (BAE), diagnostic cerebral angiogram, interventional cerebral angiogram and UAE. Diagnostic cerebral angiogram DRLs exceeded the published DRL data ranges for all parameters. Interventional cerebral angiogram exceeded published ranges for FT only. Uterine artery embolisation exceeded these ranges for KAP and K_a,r_. The 7/13 procedures with no or incomplete published DRLs to date for comparison are pigtail catheter insertion, bilateral nephrostomy, selective abdominal vessel angiogram, PICC insertion, PTBD internalisation, unilateral antegrade ureteric stent and PTC.

**TABLE 2a:** Comparison with published data on kerma air product and diagnostic reference levels.

Procedure	CHBAH, 2022	DRL range	Papanastassiou et al.^18^	Schegerer et al.^21^	Malan et al.^13^	Rizk et al.^26^	Koir et al.^14^	Etard et al.^27^	Ruiz-Cruces et al.^28^	Heilmaier et al.^29^	Erskine et al^17^	Zotovia et al.^30^
Country	South Africa	-	Greece	Europe	South Africa	Lebanon	Kenya	France	Spain	Switzerland	Australia	Bulgaria
Percutaneous transhepatic biliary drainage (PTBD)	24	23–145	53.8	23	46	145	56	33.5	30	60.5	-	56
Bronchial artery embolisation (BAE)	131	73–131.4	-	-	73	-	-	131.4	-	-	-	-
Pigtail insertion	7.5	N/A	-	-	-	-	-	-	-	-	-	-
Nephrostomy (Unilateral)	10	10–47	-	-	10	40	47	-	-	12.6	10.8	-
Nephrostomy (Bilateral)	10	N/A	-	-	9	-	-	-	-	-	-	-
Selective abdominal vessels-interventional angiogram	776	N/A	-	-	170	-	-	-	-	-	-	-
Diagnostic cerebral angiogram	209.3	55-87.5	70.2	-	55	71	-	87.5	-	-	82.6	-
Peripherally inserted central catheter (PICC)	2	N/A	-	-	-	-	-	1.2	-	-	-	-
PTBD internalisation	57	N/A	-	-	-	-	-		-	-	-	-
Uterine artery embolisation (UAE)	1463.8	118.4–214	-	-	-	-	-	174.4	214	118.4	191	-
Unilateral antegrade ureteric stent	23	N/A	-	-	-	-	-	-	-	-	-	-
Percutaneous transhepatic cholangiogram (PTC)	9	N/A	34.4	-	-	-	-	-	-	-	-	-
Interventional cerebral angiogram	275	63–233.4	-	-	63	197	-	233.5	-	-	152.9	41

*Source:* Adapted from Malan L, Pitcher RD, Da Silva M, Breuninger S, Groenewald W. Diagnostic reference levels for fluoroscopically guided procedures in a South African tertiary hospital. Acta Radiol. 2021;62(6):807–814. https://doi.org/10.1177/0284185120938371

CHBAH, Chris Hani Baragwanath Academic Hospital; DRL, diagnostic reference level; N/A, not applicable.

**TABLE 2b:** Comparison with published data on reference point air kerma and diagnostic reference levels.

Procedure	CHBAH, 2022	DRL range	Papanastassiou et al.^18^	Schegerer et al.^21^	Malan et al.^13^	Rizk et al.^26^	Koir et al.^14^	Etard et al.^27^	Ruiz-Cruces et al.^28^	Heilmaier et al.^29^	Erskine et al^17^	Zotovia et al.^30^
Country	South Africa	-	Greece	Europe	South Africa	Lebanon	Kenya	France	Spain	Switzerland	Australia	Bulgaria
Percutaneous transhepatic biliary drainage (PTBD)	13.8	195–1406	399.8	195	227	1406	320	253	-	580	320	-
Bronchial artery embolisation (BAE)	343	259–827	-	-	259	-	-	827	-	-	-	-
Pigtail insertion	37	N/A	-	-	-	-	-	-	-	-	-	-
Nephrostomy (Unilateral)	26	63–412	-	-	63	412	-	-	-	80	245	-
Nephrostomy (Bilateral)	62	N/A	-	-	56	-	-	-	-	-	-	-
Selective abdominal vessels-interventional angiogram	2227.8	N/A	-	-	877	-	-	-	-	-	-	-
Diagnostic cerebral angiogram	868.5	289–628	494.0	-	289	596	-	628	-	-	-	-
Peripherally inserted central catheter (PICC)	5	1.0–4.0	-	-	-	-	-	4	1	-	-	-
PTBD internalisation	259	N/A	-	-	-	-	-	-	-	-	-	-
Uterine artery embolisation (UAE)	4019	729–1240	-	-	-	-	-	729	168.9	1240	-	-
Unilateral antegrade ureteric stent	118.5	N/A	-	-	-	-	-	-	-	-	-	-
Percutaneous transhepatic cholangiogram (PTC)	28.5	N/A	194.0	-	-	-	-	-	-	-	-	-
Interventional cerebral angiogram	1744	505–2993.5	-	-	505	2583	-	2993.5	-	-	-	-

*Source:* Adapted from Malan L, Pitcher RD, Da Silva M, Breuninger S, Groenewald W. Diagnostic reference levels for fluoroscopically guided procedures in a South African tertiary hospital. Acta Radiol. 2021;62(6):807–814. https://doi.org/10.1177/0284185120938371

CHBAH, Chris Hani Baragwanath Academic Hospital; DRL, diagnostic reference level; N/A, not applicable.

**TABLE 2c:** Comparison with published data on fluoroscopy time and diagnostic reference levels.

Procedure	CHBAH, 2022	DRL range	Papanastassiou et al.^18^	Schegerer et al.^21^	Malan et al.^13^	Rizk et al.^26^	Koir et al.^14^	Etard et al.^27^	Ruiz-Cruces et al.^28^	Heilmaier et al.^29^	Erskine et al^17^	Zotovia et al.^30^
Country	South Africa	-	Greece	Europe	South Africa	Lebanon	Kenya	France	Spain	Switzerland	Australia	Bulgaria
Percutaneous transhepatic biliary drainage (PTBD)	6.2	10–22.9	22.9	10	20	20	23	15.7	17.3	-	-	12.2
Bronchial artery embolisation (BAE)	33.5	37.4–38	-	-	38	-	-	37.4	-	-	-	-
Pigtail insertion	2.4	N/A	-	-	-	-	-	-	-	-	-	-
Nephrostomy (Unilateral)	3.4	3.5–18	-	-	4	11	18	-	-	-	3.5	-
Nephrostomy (Bilateral)	6.3	N/A	-	-	4	-	-	-	-	-	-	-
Selective abdominal vessels-interventional angiogram	28.3	N/A	-	-	29	-	-	-	-	-	-	-
Diagnostic cerebral angiogram	28.4	8–10.3	9.2	-	14	8	-	10.3	-	-	6.25	-
Peripherally inserted central catheter (PICC)	4	N/A	-	-	-	-	-	1	-	-	-	-
PTBD internalisation	16.7	N/A	-	-	-	-	-	-	-	-	-	-
Uterine artery embolisation (UAE)	24.8	28.7–31	-	-	-	-	-	28.7	31	-	-	-
Unilateral antegrade ureteric stent	15.2	N/A	-	-	-	-	-	-	-	-	-	-
Percutaneous transhepatic cholangiogram (PTC)	0.7	N/A	14.2	-	-	-	-	-	-	-	-	-
Interventional cerebral angiogram	34.1	9.2–32	-	-	25	28	-	62.9	-	-	32	9.2

*Source:* Adapted from Malan L, Pitcher RD, Da Silva M, Breuninger S, Groenewald W. Diagnostic reference levels for fluoroscopically guided procedures in a South African tertiary hospital. Acta Radiol. 2021;62(6):807–814. https://doi.org/10.1177/0284185120938371

CHBAH, Chris Hani Baragwanath Academic Hospital; DRL, diagnostic reference level; N/A, not applicable.

The procedure type with the most DRL published data were PTBD with published DRLs from 9/10 studies reviewed. Dose parameter with the most published data was KAP with 34 DRLs across all procedures. Regarding published DRLs, the highest recorded across all parameters was for interventional cerebral angiogram (KAP-233.5 Gy/cm^2^, K_a,r_–2993.5 mGy and FT-62.9 min). The lowest was for PICC insertion (KAP-1.2 Gy/cm^2^, K_a,r_–1 mGy and FT-1).

## Discussion

The study identified the current scope of IR procedures carried out at a single institution while simultaneously determining procedures not performed or those performed with low frequency. Hepatobiliary (liver and pancreas) IR has room for growth through adding procedures such as TIPS, portal vein embolisation, transarterial chemo-embolisation (TACE), radio-embolisation with radioactive microspheres and radiofrequency ablation, a similar outcome to what another local study^13^ at a public sector hospital found. This reflects the limited IR expertise in public sector hospitals and should improve with time as more radiologists and registrars are trained in IR.

Uterine artery embolisation recorded the highest DAP doses (selective abdominal vessel interventional angiogram was the second highest), in keeping with findings from other studies. Based on published literature, abdominal and pelvic angiographic interventions^13,17^ typically have the highest recorded DAP.

This study showed a relationship between KAP and K_a,__r_. Generally, high KAP corresponds to high K_a,r_. The relationship between KAP and FT is not as strong. This is consistent with findings from local^13^ and international^17,18^ studies and emphasises that no dose parameters can be extrapolated to infer radiation exposure; instead, as many parameters as possible should be evaluated when optimising radiation exposure. This is echoed in the recommendations by the ICRP.^10^

Unilateral percutaneous nephrostomy (PCN) had the widest variation in KAP (75th:50th percentile ratio of 5) and wide, although not the widest, variation in K_a,r_ (1.8) and FT (2.1). This is consistent with findings from other published studies.^13^ As with the other institutions, nephrostomies are entry-level procedures performed by mostly junior registrars, and the wide variation in dose is most likely attributable to the inconsistency in skill level. The effect of operator experience level on radiation dose has been studied extensively.^19,20^

Regarding neurological procedures, there was no consistency in the degree of dose variation. Diagnostic cerebral angiogram had the narrowest variation in KAP and K_a,r_, whereas interventional cerebral angiogram had the second widest variation in dose. Both these procedures were performed by two highly experienced specialist radiologists. This shows that dose variation is a factor of more than just skill level but rather several factors, including but not limited to patient factors and lesion or pathology factors. Many studies have assessed the effect of the degree of lesion complexity on patient radiation.^18,21,22,23^ This study could not incorporate lesion complexity because it was a retrospective study and such information was neither recorded, nor was there a standardised way of assessing complexity for the procedures. Diagnostic reference level data from studies that did not include lesion complexity are still of great value.^24^

This study did not use patient weight as a dose parameter. The link between body mass index (BMI) and patient radiation dose has long been established for digital radiography and fluoroscopically-guided injections.^23,25^ Regarding IR, some studies found that stratification by weight had no statistically significant effect on third quartile values (DRLs) for head procedures but had significant effect on body procedures.^22,24^

Diagnostic cerebral angiogram (all parameters), interventional cerebral angiogram (FT) and UAE (KAP and K_a,r_) exceeded published local and international DRL ranges. As such, these procedure types need to be reviewed and optimised in accordance with the recommendations of the ICRP so that they are aligned with published DRLs.^10^ This study is a step towards establishing national IR DRLs, adding to the work by Malan et al.^13^

### Limitations

As a retrospective study, a limited number of parameters that impact dose were evaluated. Parameters such as patient weight (body mass index [BMI]) and lesion complexity could not be included, as this information was not available. Going forward, such information should be recorded to improve the quality of future studies. Although the number of cases for calculating DRLs with a reasonable 95% confidence interval as suggested by Miller et al.^24^ is 30, the ICRP recommends anything above 15, which is what was applied in this study.

### Recommendations

There is a need for standardisation of the terminology used for IR procedures. As with Malan et al.,^13^ this study stratified nephrostomy into unilateral and bilateral, whereas most other studies did not. In addition, this study also recognises antegrade ureteric stents because it is a procedure that can be performed post nephrostomy. The same principle applies to PTBD internalisation. Standardisation will enable more accurate comparison of dose. Pertaining to the institution, DRLs should be evaluated annually to keep up with factors such as operator expertise, changing procedure profile and machines.

## Conclusion

This study is in line with international radiation protection initiatives. At an institution level, it contributes to patient radiation optimisation and nationally to establishing national interventional radiology DRLs. The LDRLs for diagnostic cerebral angiogram, interventional cerebral angiogram and UAE exceeded international ranges in this study. These procedures must be reviewed for radiation optimisation. A suggestion is to include patient weight and complexity of lesions as input parameters.
